# Plasmonic IQ modulators with attojoule per bit electrical energy consumption

**DOI:** 10.1038/s41467-019-09724-7

**Published:** 2019-04-12

**Authors:** Wolfgang Heni, Yuriy Fedoryshyn, Benedikt Baeuerle, Arne Josten, Claudia B. Hoessbacher, Andreas Messner, Christian Haffner, Tatsuhiko Watanabe, Yannick Salamin, Ueli Koch, Delwin L. Elder, Larry R. Dalton, Juerg Leuthold

**Affiliations:** 1 Institute of Electromagnetic Fields (IEF), ETH Zurich, 8092 Zurich, Switzerland; 2 Department of Chemistry, University of Washington, Seattle, WA 98195-1700 USA

## Abstract

Coherent optical communications provides the largest data transmission capacity with the highest spectral efficiency and therefore has a remarkable potential to satisfy today’s ever-growing bandwidth demands. It relies on so-called in-phase/quadrature (IQ) electro-optic modulators that encode information on both the amplitude and the phase of light. Ideally, such IQ modulators should offer energy-efficient operation and a most compact footprint, which would allow high-density integration and high spatial parallelism. Here, we present compact IQ modulators with an active section occupying a footprint of 4 × 25 µm × 3 µm, fabricated on the silicon platform and operated with sub-1-V driving electronics. The devices exhibit low electrical energy consumptions of only 0.07 fJ bit^−1^ at 50 Gbit s^−1^, 0.3 fJ bit^−1^ at 200 Gbit s^−1^, and 2 fJ bit^−1^ at 400 Gbit s^−1^. Such IQ modulators may pave the way for application of IQ modulators in long-haul and short-haul communications alike.

## Abstract (web summary)

Increasing bandwidth demands in optical communications requires components to be compact with energy-efficient operation. Here, the authors demonstrate plasmonic IQ modulators on a silicon photonics platform with phase shifters, operating with sub-1V electronics at 100 GBaud and low electrical energy consumption.

## Introduction

Electro-optic modulators that deal with highest data rates on smallest footprints while being energy efficient^1–8^ are urgently needed to cope with the rapidly growing bandwidth requirements of future communication networks^9–13^. Particularly, cloud-based services drive an annual traffic growth of 60%, with data centers reaching 80,000 servers^10^. In recent years, coherent communications has emerged as a solution offering the highest data transmission capacity and spectral efficiency^14–16^. It actually is one of the few schemes with the potential to satisfy the ever-growing bandwidth demands of today’s information society^10^. However, coherent communications requires in-phase/quadrature (IQ) modulators^15,17,18^, which allow encoding of information onto the phase and the amplitude of light. Ideally, such modulators should feature high-speed, offer a compact fooptrint, and operate energy efficiently. And while IQ modulators have already convincingly demonstrated operation at highest speed^19–21^, they are still too bulky to be a convincing solution for short-reach communication systems of the future. If these modulators would be energy-efficient, cost-efficient and densely integrated on a chip, they could find significantly broader applications. They might find deployment in applications ranging from classical long-haul communications to the rapidly growing, capacity-hungry inter-datacenter and intra-datacenter communications^11^, to access networks^22,23^, and potentially even in chip-to-chip communications and data transfer between cores of multi-core processors^13^.

To evaluate compactness, energy efficiency, and speed of an electro-optic modulator, the voltage-length product and the achievable line rate have emerged as useful Figures of Merit (FoMs). The voltage-length product *U*_π_*L* defines the device length required to switch from the on- to the off-state with 1 V, which is a convenient measure to evaluate compactness as well as prospects for dense integration and parallelization. The most successful IQ modulator technology to date is based on lithium niobate (LiNbO_3_). It is the standard in any long-haul optical communication system^24^ and offers line rates up to 600 Gb s^−1^ per wavelength and polarization^20,25^ with fiber-to-fiber losses around 10 dB. Nevertheless, LiNbO_3_ devices have typical length in the order of cm (*U*_π_*L* ≈ 1–10 V cm, active length ≈ 1–10 cm), which hampers any dense integration and ergonomic parallelization. Conversely, indium phosphide (InP) modulators have been identified as a promising and more compact solution (*U*_π_*L* ≈ 1 Vcm, active length ≈ 3.6 mm)^26^. They offer similar line rates of up to 660 Gb s^−1^^19,21,27^, with fiber-to-fiber losses around 9 dB. However, these devices rely on more demanding fabrication technologies and lack the intriguing prospect of monolithic co-integration with CMOS electronics. The silicon platform in contrast promises the co-integration of electronics and photonics on a single chip^4,28^. Silicon photonic (SiP) IQ modulators offer relatively compact footprints. They have typical lengths of a few mm (*U*_π_*L* ≈ 1 Vcm^26,29^, active length ≈ 4.5 mm), and already have operated up to 350 Gb s^−1^ with fiber-to-fiber losses of around 15 dB^30^. By introducing organic electro-optic (OEO) materials to the SiP platform^31–33^ even shorter devices with 0.5-mm-long active sections (*U*_π_*L* ≈ 0.5–1 Vmm^32,33^) and line rates of up to 400 Gb s^−1^ ^33^ became possible with fiber-to-fiber losses of around 17.5 dB^33^. Even though all of these platforms offer highest line rates, the dimensions of these devices are still relatively large. The photonic nature of these devices and the traveling wave electrode designs required for high-speed operation ultimately limit dense integration capabilities. Supplementary Table 1 summarizes the performance of state-of-the-art IQ modulators.

In addition to speed and compactness, drive voltages compatible with CMOS levels (≤1 V) and small device capacitances are required in order to keep electronics and on-chip heat management relaxed. These requirements can be expressed by the energy that an electrical driver has to provide to modulate one bit, *E*_bit_^34^, which ideally would be below a femtotjoule per bit (fJ bit^−1^). While intensity modulators have already demonstrated sub-fJ bit^−1^ modulation^8,35,36^ only few have shown to operate with lowest energy consumption at highest-speed (70 Gbit s^−1^)^8^.

Recently, plasmonic approaches have emerged as a promising alternative^37^. The success of these schemes relies on a combination of plasmonic slot-waveguides and an organic^38^ or ferroelectric^39^ nonlinear material. These modulators promise lowest voltage-length products (*U*_π_*L* ≈ 40 V µm)^40^, ultra-fast electro-optic modulation featuring an extraordinary electro-optical bandwidth beyond 500 GHz^41–43^ and modulation up to the THz^44^, and a most compact footprint^5,40^. The small size has already enabled dense modulator arrays for interconnect applications demonstrated on a microscopic footprint^45,46^. And while plasmonics has already shown compact footprints, and low electrical energy consumptions^47^, it is not clear if the technology can also offer lowest-drive voltages with sub-fJ bit^−1^ electrical energy consumptions at highest-speed.

In this paper, we demonstrate compact plasmonic IQ modulators on silicon with an active section occupying as little as 4 × 25 µm × 3 µm of footprint (four phase modulators with a footprint of 25 µm × 3 µm each) and show stable operation at data rates up to 400 Gb s^−1^ with a small *U*_π_*L* ≈ 130 V µm. Furthermore, the devices operate with sub-1 V driving electronics at 100 GBd. Since the devices feature capacitances of a few femtofarad only, this technology features among the lowest electrical energy consumption of any IQ modulator technology demonstrated to date. We calculate electrical energy consumptions to be as low as 0.07 fJ bit^−1^ at 50 Gb s^−1^, 0.3 fJ bit^−1^ at 200 Gb s^−1^, and 2 fJ bit^−1^ at 400 Gb s^−1^. This new IQ modulator implementation takes advantage of the combination of silicon photonics^48,49^, organic electro-optics^50,51^, and plasmonic sub-wavelength light confinement^52–55^, all of which allow for efficient electro-optic modulation on micrometer footprints. In detail, organic electro-optics and plasmonics offer compact footprints and high-speed modulation. By combining these high-speed modulators with silicon photonic thermo-optic phase shifters, low-loss waveguides, and on-chip power splitters we are able to show highest-speed modulation with lowest drive voltages, high extinction ratios and lowest electrical energy consumptions. The content of this work is in part based on preliminary results first presented at the CLEO conference^56^.

## Results

### Plasmonic IQ modulators

**Fig. 1:**
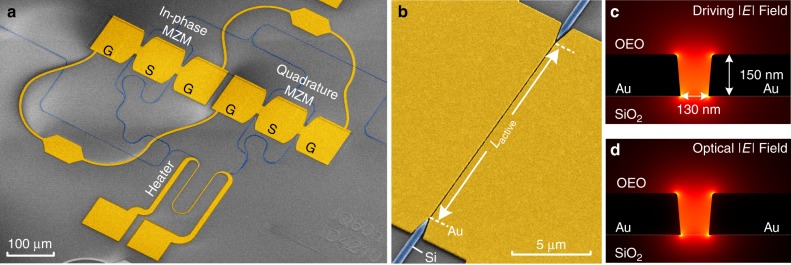
Plasmonic IQ modulator on the silicon platform. **a** Colorized scanning electron microscope picture of a plasmonic IQ modulator consisting of two Mach-Zehnder modulators (MZMs). **b** Zoom into the active section of the modulator, a plasmonic slot waveguide. Light from silicon (Si) feeding waveguide is coupled to the gold (Au) slot waveguide by tapered mode converters. The slot is filled with an organic electro optic (OEO) material (not depicted). **c**, **d** Cross section of the plasmonic slot waveguide with simulated **c** electrical driving field and **d** optical field

The plasmonic-organic hybrid IQ modulators discussed here, consist of two imbalanced high-speed plasmonic Mach-Zehnder modulators (MZMs)^57^ integrated into a SiP Mach-Zehnder interferometer (MZI), see Fig. 1a. The phase delay between I and Q branches can be adjusted either by a thermo-optic phase shifter (heater) or by tuning the wavelength, enabled by the imbalance in the SiP MZI. DC biases are used to independently adjust the operating points of the two MZMs. The active sections of the MZMs consist of 15 or 20 µm long plasmonic slot waveguides^58^ with a slot width of 130 nm, Fig. 1b. The metal-insulator-metal (MIM) plasmonic slot waveguide is filled with the organic electro-optic (OEO) material composite HD-BB-OH/YLD124^59^. Within the active section, both the electrical RF driving field and the optical field are tightly confined to the plasmonic slot and are overlapping nearly perfectly, Fig. 1c, d.

The linear electro-optic effect in the OEO material allows efficient encoding of the electrical driving signal onto the optical carrier^3^. The devices feature voltage-length products of *U*_π_*L* ≈ 130 V µm and on-chip optical losses of 11.2 dB for the device with 15 μm and 13.7 and 14.5 dB for the devices with 20 μm length. The on-chip losses can be attributed to photonic-plasmonic conversion losses of ~ 1.7 dB per converter and phase shifter losses of ~0.5 dB µm^−1^. These losses are higher than what was expected from previous fabrication runs (~0.5 dB per converter, 0.38 dB µm^−1^ ^38^). In addition, we measure fiber-to-chip coupling losses of 4 dB per coupler. With optimized fabrication and coupling techniques, fiber-to-fiber losses below 10 dB should already now be possible. By further exploiting the design freedom offered by organic electro-optics, higher in-device nonlinearies may be expected^60^. Ultimately, fiber-to-fiber losses below 6 dB should become practical, see Methods section.

### High-speed data modulation with driving amplifiers

**Fig. 2:**
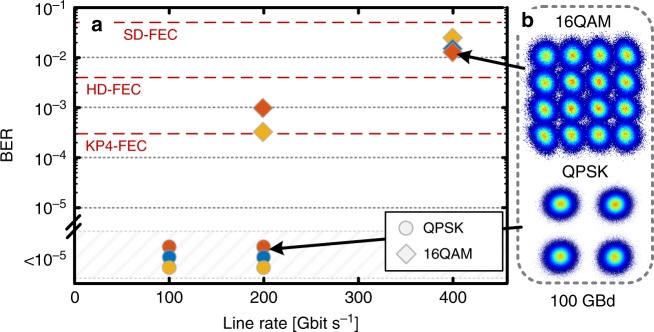
Results of the high-speed data modulation experiments. **a** BER as a function of the line rate for three devices, IQ1-3 (blue, red, and yellow). QPSK: circles, 16QAM: diamonds. Operation well below the KP4 FEC threshold for all QPSK measurements. Operation below the 7% HD-FEC threshold at 50 GBd 16QAM and the SD-FEC limit 100 GBd 16QAM. Measurements with BERs below 1 × 10^−5^ are grouped. **b** Constellation diagrams for 100 GBd 16QAM and QPSK

The plasmonic IQ modulators introduced here offer high line-rate complex modulation up to 400 Gb s^−1^ on a compact footprint. The active sections occupy only 4 × 25 µm × 3 µm, the contacting electrodes for easy testing consume ~400 µm × 600 µm—but are not needed if cointegrated with electronics. The devices were tested for high-speed signal generation at 50 GBd and 100 GBd, each with QPSK and 16QAM. This corresponds to line rates from 100 Gb s^−1^ up to 400 Gb s^−1^. Figure 2 plots the measured bit error ratios (BERs) for three investigated devices IQ1–IQ3 (see Supplementary Table 2) as a function of the line rate. For QPSK modulation, all devices showed BERs well below the KP4 forward error correction (FEC) limit of 2 × 10^−4^ at both 50 and 100 GBd^61^. For 16QAM modulation, BERs are well below the HD-FEC limit of 3.8 × 10^−3^ at 50 GBd and below the SD-FEC limit of 4 × 10^−2^ at 100 GBd (400 Gbit s^−1^)^25^. At 400 Gbit s^−1^ the device IQ3 dissipates an electrical energy as low as 2 fJ bit^−1^ when operated with a driving signal of *U*_meas50Ω,pp_ = 1.48 V. The voltage is provided by an electrical driving amplifier and has been measured using a 50 Ω oscilloscope. Measurements displayed in Fig. 2 are summarized in Supplementary Table 3.

Furthermore, we test the compatibility of plasmonic IQ modulators in systems without electronic equalization. Table 1 summarizes the performed measurements. We found BERs below the KP4 FEC limits for at 50/100 GBd QPSK and below the SD-FEC limit at 50 GBd 16QAM. At 100 GBd, a static digital post distortion was applied to account for the strong low-pass characteristics of the electronic components. The experimental setup and the used DSP can be found in the Methods section.

**Table 1:** Device operation without electronic equalization

	(a) 16 QAM 100 GBd	(b) QPSK 100 GBd	(c) 16 QAM 50 GBd	(d) QPSK 50 GBd
Line rate	400 Gb s^−1^	200 Gb s^−1^	200 Gb s^−1^	100 Gb s^−1^
Const diagr.				
BER	4.5 × 10^−2^	6.5 × 10^−5^	6.8 × 10^−3^	1.9 × 10^−9^

(a) 100 GBd 16QAM and (b) QPSK. Static digital post distortion (DPD) was applied to correct the low-pass characteristics of the electronic components. (c) 50 GBd 16QAM and (d) QPSK. No DPD was applied at 50 GBd

### High-speed data modulation with sub-1 V driving electronics

Plasmonic IQ modulators offer high-speed data modulation without the use of expensive and energy-hungry electrical RF amplifiers. Sub-1 V driving electronics in combination with device capacitances in the range of ~3 fF allows for sub-fJ bit^−1^ electrical energy consumptions up to 200 Gb s^−1^. The electrical energy efficiency of a modulator is determined by its drive voltage *U*_pp_ and device capacitance *C*. It can be estimated to be proportional to *CU*_pp_^2^, derived in the ref. ^62^ and adapted for QAM modulation formats in the ref. ^40^. Note that the voltage drop *U*_IQ,pp_ across the plasmonic IQ’s active section is estimated to be twice the voltage measured with a 50 Ω oscilloscope, hence, *U*_pp,IQ_ *≈* 2*U*_pp,meas50Ω_, see Methods section. Neither heaters nor driving amplifiers were used to operate the device.

Table 2 shows selected constellation diagrams at (a) 100 GBd, (b, c) 50 GBd, and (d) 25 GBd, all operated with sub-1 V driving electronics. At 200 Gb s^−1^ the device was operated with a drive voltage of *U*_meas50Ω,pp_ = 567 mV, resulting in an electrical energy consumptions of 0.3 fJ bit^−1^. At 50 Gb s^−1^, the device was operated with *U*_meas50Ω,pp_ = 145 mV resulting in an electrical energy consumption of 0.07 fJ bit^−1^. The slight asymmetry that can be seen in the constellation diagrams of the QPSK signals (a) and (d) is due to an imperfect IQ angle and led to a small BER penalty. Please note that the total system energy consumption is strongly influenced by the transmitter (DAC, DC sources, clock source, laser source), and receiver components (photodiodes, optical amplifiers, oscilloscope) and is strongly defined by the energy efficiencies of these components. Measurements performed with low driving voltages are summarized in Supplementary Table 4.

**Table 2:** Device operation with sub-1 V driving electronics without driving amplifier

	(a) QPKS 100 GBd	(b) 16QAM 50 GBd	(c) QPSK 50 GBd	(d) QPSK 25 GBd
Line rate	200 Gbit s^−1^	200 Gbit s^−1^	100 Gbit s^−1^	50 Gbit s^−1^
Const diagr.				
BER	1.4 × 10^−3^	2.0 × 10^−2^	2.0 × 10^−4^	2.0 × 10^−3^
*U*_meas50Ω,pp_	426 mV	567 mV	326 mV	145 mV
*E*_bit_	0.61 fJ bit^−1^	0.30 fJ bit^−1^	0.36 fJ bit^−1^	0.07 fJ bit^−1^

Constellation diagrams and sub-fJ bit^−1^ electrical energy consumptions at (a) 100 GBd, (b, c) 50 GBd, and at (d) 25 GBd

### Stable device operation

**Fig. 3:**
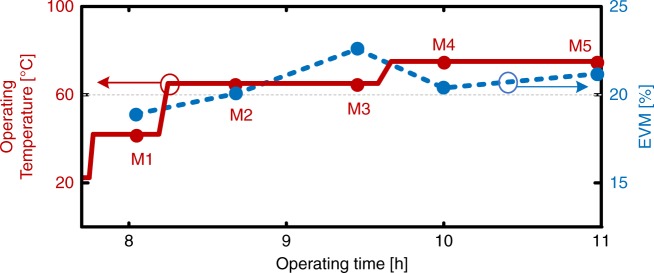
Optical modulation at elevated temperatures for the device IQ1. Operation temperature (solid red) and EVM (dashed blue) as a function of the operating time. The EVM remains nearly constant and stays around 20% for all temperatures. All measurements at 50 GBd QPSK. More information on measurements M1–M5 can be found in the Supplementary Table 5

Plasmonic IQ modulators offer reliable and stable operation. We demonstrate stable operation for >10 h as well as operation at elevated temperatures of up to 75 °C. The plasmonic IQ modulator IQ1 (see Supplementary Table 2) was operated for ~8 h before we started testing at elevated temperatures. To perform experiments at higher temperatures, the device was mounted on a temperature-controlled sample stage. The sample stage was then heated to the desired temperature. The surface temperature of the stage was measured, see Methods section. Due to the optical power in the slot the temperature in the slot is expected to exceed the set value. However, the temperature in the slot is expected to be at least ~30 °C below the materials glass transition temperature, as no depoling was observed during the measurements. Figure 3 depicts the operating temperature and error vector magnitudes (EVM) taken at five different measurement points M1–M5 (circles) during the operating time of this experiment. After a reference measurement at 42 °C was performed (measurement M1), the device was heated and operated for 70 min at 65 °C and for 80 min at 75 °C. Test measurements M1–M5 have been recorded during this time span. We observe the expected burn-in within the first ~30 min at 60 °C, the EVM increases from 18 to 20%. This originates from a slight increase of the modulator’s U_*π*_*L* product. During this burn-in process the OEO molecules relax to a stable orientation, after which a stable device performance is found. No further degradation of the modulation performance was observed during the temperature tests, the EVM stayed around 20%. More information on the operation at elevated temperatures can be found in the Methods section.

We identified ~75 °C as the upper temperature limit for stable device operation for the OEO material used in this work. While device IQ1 was operating stably at this temperature during and after the test, we observed de-poling of device IQ3. This temperature of 75 °C is slightly below the temperature of ~80 °C where we noticed de-poling in an earlier experiment^5^. At these temperatures even small variations in temperature and optical power may induce de-poling, which typically occurs around 30 °C^63–65^ below the OEO material’s glass transition temperature *T*_g_ (in this work, *T*_g_ ~ 110 °C)^59^. It is important to note that high-*T*_g_ and cross-linked OEO materials potentially offer much better long-term stability at higher temperatures.^51^^,^^66^^,^^67^

## Discussion

We have presented compact IQ modulators with an active section occupying 4 × 25 µm × 3 µm operating with drive voltages below 1.5 V at up to 400 Gb s^−1^. We furthermore show high-speed operation of the modulators with sub-1 V driving electronics. We demonstrate low electrical energy consumptions of 0.07 fJ bit^−1^ at 50 Gb s^−1^, 0.3 fJ bit^−1^ at 200 Gbit s^−1^. At 400 Gb s^−1^ and a drive voltage of ~1.5 V we find an electrical energy consumption of 2 fJ bit^−1^ These devices have optical on-chip losses between 11 and 15 dB, demonstrate stable performance (tested for >10 h), and operation at elevated temperatures up to 75 °C. Due to the compactness and energy-efficiency of our modulators, they can be easily parallelized on a tiny footprint at low cost.

The plasmonic modulator technology is a solution that can meet the energy and footprint needs of future optical communications systems. It enables highly parallelized, high-speed coherent communications on a most compact footprint. With this technology, coherent optical communications may become accessible to a much broader field of applications ranging from inter/intra datacenter communications all the way up to access and backbone networks^11,22,23^. Potentially, considering advances in fabrication technology and organic electro-optics, these devices may be envisioned to even find applications in chip-to-chip communications and communications between cores of multi-core processors^13^.

## Methods

### Devices

Within this work, three similar devices (IQ1–IQ3) have been investigated. They feature the properties given in Supplementary Table 2. Losses were measured at maximum transmission. On-chip silicon photonic losses as well as fiber-to-chip coupling losses have been determined by silicon photonic reference structures and photonic cutback structures on the same chip. Plasmonic propagation losses have been determined by plasmonic cutback measurements. Photonic-to-plasmonic converter losses have been derived indirectly from the loss contributions of the individual components.

Taking into account these losses, the maximum optical power within one slot was 8 dBm. In the plasmonic section, light propagates well confined to the slot waveguide. No crosstalk originating from plasmons propagation along the top surface of the gold electrodes can be observed.

Note that both the fiber-to-fiber and the on-chip loss leave room for improvement. By relying on dedicated fabrication processes and the use of established coupling techniques, fiber-to-fiber losses of below 10 dB should be within reach.

By switching to established coupling techniques of the industry, the fiber-to-fiber losses can be reduced by 5 dB to ~14.2 dB. Our current fiber-to-chip coupling of ~4 dB per coupler is higher than what is offered by the industry. SiP foundries offer ~2.5 dB per coupler^68^. Blazed GCs similar to the ones used within this work, but optimized for perpendicular incidence offer ~1.5 dB per coupler^69^. Standard edge coupling offers similar losses of ~1–1.5 dB per coupler^68^.

Furthermore, the photonic-to-plasmonic converters (PPCs) used in this work were optimized for slot widths of 80 nm instead of 130 nm and show higher losses than expected. These linearly tapered PPCs introduce losses of ~1.7 dB per converter while ~0.5 dB per converter are possible with optimized structures. These more efficient converters would improve the fiber-to-fiber losses by another 2.4 dB to ~11.8 dB. Plasmonic propagation losses were estimated using four IQ modulators with two different lengths to be 0.5 dB µm^−1^, being in good agreement with reference structures on the same chip. In previous experiments, such losses of 0.5 dB µm^−1^ have been found in 80 nm wide slots^38,57^, while slot widths of 130 nm should result in values below 0.38 dB µm^−1^ ^38^. We attribute these increased losses compared to previous runs to higher roughness and impurity of gold, deposited in a multi-user and multi-material deposition system. Switching to a dedicated deposition tool should reduce the fiber-to-fiber losses further to 9.9 dB. Alternatively, a higher gold quality would permit reduction of the slot width to 80 nm, while keeping the losses the same. This would reduce the voltage-length product from *U*_π_*L* ≈ 130 V µm by a factor of approximately two to *U*_π_*L* ≈ 65 V µm. Hence, with current fabrication techniques and coupling options sub-10 dB fiber-to-fiber losses are within reach.

Additionally, the plasmonic-organic hybrid modulator technology offers an intriguing route to improve performance without altering the device structure: the technology can be used with alternative, higher performance OEO materials that have been recently been developed or proposed. While in the current experiment, in-device nonlinearities of ~160 pm V^−1^ are achieved, higher in-device nonlinearity EO materials with good thermostability are within reach. A doubling of the EO coefficient from ~160 pm V^−1^ with the current material to 320 pm V^−1^ (recently demonstrated in-device nonlinearity: 390 pm V^−1^)^70^, would allow to reduce the length of the device by a factor of two, potentially reducing the fiber-to-fiber losses to 6 dB. Because the required drive voltage of a plasmonic MZM depends linearly on the length of the plasmonic section and the nonlinearity of the OEO material^38^, a doubling of the material’s nonlinearity would allow for a 50% shorter device, while keeping the drive voltage constant. Alternatively, the drive voltage could be reduce by a factor of two.

Optimizing OEO materials for plasmonic applications promises further improvements with nonlinearities of >1000 pm V^−1^ ^60,71^ and consequently will allow to further reduce the fiber-to-fiber loss and the voltage-length product.

### Energy consumption per bit and drive voltage

The energy consumption per bit *E*_bit_ has emerged as a figure-of-merit (FOM) to compare different electro-optic modulator technologies^62^. It depends mainly on the device impedance (capacitance) and the electrical drive voltage. Low *E*_bit_ values indicate that the devices can be operated with energy efficient electrical driver circuits.

The *E*_bit_ FOM of a purely capacitive Mach-Zehnder modulator operated with an NRZ signal is given by^35,62^ $E_{{\mathrm{bit}}} = 1/4 \cdot C_{{\mathrm{dev}}}U_{{\mathrm{pp}}}^2{\mathrm{/bit}}$, where *C*_dev_ is the device capacitance and *U*_pp_ is the peak-to-peak drive voltage. For complex modulation formats such as QPSK and 16QAM:^40^

1 $$E_{{\mathrm{bit}},{\mathrm{QPSK}}}\,=\,1/4 \cdot C_{{\mathrm{MZM}}}U_{{\mathrm{pp}}}^2/{\mathrm{bit}},$$

2 $$E_{{\mathrm{bit}},16{\mathrm{QAM}}}\,=\,5/72 \cdot C_{{\mathrm{MZM}}}U_{{\mathrm{pp}}}^2/{\mathrm{bit}},$$

where *C*_MZM_ is the capacitance of a single MZM.

The electrical peak-to-peak voltage *U*_pp_ applied to the device can be measured using an electrical oscilloscope. Most driving electronics and RF measurement equipment have an impedance of 50 Ω. However, the plasmonic modulators are purely capacitive^72^. When applied to a standard 50 Ω source, twice the 50 Ω-specified voltage can be utilized by the capacitive device^35,72^. For calculation of *E*_bit_ we consider twice the measured voltage *U*_pp_ = 2*U*_meas50Ω,pp_. We do not take the frequency dependent RF-probe loss into account amounting to between ~0.25 dB at 7 GHz and ~1.1 dB at 67 GHz. Therefore, the effective drive voltages applied to the device is slightly lower than the values in this manuscript.

**Fig. 4:**
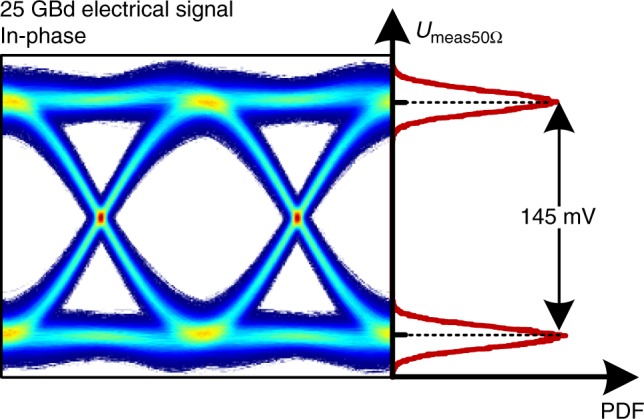
Measured electrical eye diagram and the probability density function (PDF). The PDF represents the voltage level distribution at the maximum eye opening where the peak-to-peak electrical drive voltage *U*_meas50Ω,pp_ is measured

Figure 4 shows a measured electrical eye diagram used to determine the electrical drive voltage *U*_meas50Ω,pp_. It shows the in-phase component of a 25 GBd QPSK signal. The peak-to-peak drive voltage *U*_meas50Ω,pp_ is measured using the probability density function (PDF) at the maximum eye opening. In this example, it is measured to *U*_meas50Ω,pp_ = 145 mV. This measurement was performed for in-phase and quadrature signals for all electrical driving signals used in this work.

### Device capacitance

The modulator impedance was determined with and without RF contact pads using frequency domain simulations in CST microwave studio. We follow the method published in ref. ^72^, where simulations and experimental verifications can be found. For the simulation without RF contact pads, the MZM was modeled as a 1 µm wide strip separated by narrow gaps from two ground planes on either side, see Fig. 2.14 and Table 1 in ref. ^72^. The device impedance was calculated from the simulated reflection coefficient *S*_11_ at the device input using $Z_{\mathrm{d}} = Z_{\mathrm{L}}(1 + S_{11})/(1 - S_{11})$ with *Z*_L_ = 50 Ω. The capacitance was determined from the simulated imaginary part of *Z*_d_ with $C_{\mathrm{d}} = - \frac{1}{{{\mathrm{Im}}\left\{ {Z_{\mathrm{d}}2\pi f} \right\}}}$, yielding *C*_d_ = 3.4fF at 20 GHz (IQ1: 2.6 fF). When including the electrical contact pads, the capacitance yielded $C_{\mathrm{d}} = 36\,{\mathrm{fF}}$. Even if the large pads are added, the energy consumption is below 1 fJ bit^−1^ at 50 Gb s^−1^ and below 5 fJ bit^−1^ at 200 Gb s^−1^.

### High-speed experiments with driving amplifiers

**Fig. 5:**
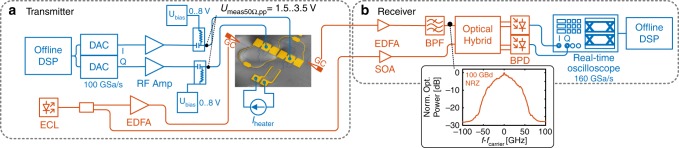
Experimental setup for high-speed data generation experiment with driving amplifier

The high-speed experiments were performed using the setup depicted in Fig. 5. At the transmitter, electrical signals (100 GBd NRZ and 50 GBd NRZ, QPSK and 16QAM) are generated offline and are sent to two (one for in-phase (I) and one for quadrature (Q) component) digital-to-analog converters (DAC, Micram DAC4, 100 GSa s^−1^, 3 dB BW: 40 GHz, vertical resolution of 6 bit). Subsequently, the electrical signals were amplified by electrical driving amplifiers (RF amps: 3 dB BW: 55 GHz or 3 dB BW: 65 GHz) before they were combined with a DC bias to control the operating points of the MZMs (between 0 and 8 V) using bias tees (3 dB BW: 65 GHz). The electrical signals are fed by two RF probes (ground-signal-ground (GSG) configuration, 1 dB BW: ~60 GHz) to the device under test (DUT). The IQ bias was adjusted by using the thermo-optic phase shifter, with an electrical heating power between 0 and ~20 mW.

Light from an external cavity laser (ECL) (wavelength around 1550 nm) was amplified to 15 dBm – 20 dBm using an erbium-doped fiber amplifier (EDFA) and was fed to- and from the chip using fiber-to-chip grating couplers. The modulated light is coupled back to fiber and send to the optical coherent receiver. At the receiver the signal is amplified by and EDFA and mixed with the local oscillator in a 90° optical hybrid. Two balanced photo diodes (3 dB BW: 70 GHz) receive the signal which is digitized by a real-time oscilloscope (160 GSa s^−1^ 3 dB BW: 63 GHz).

### High-speed data modulation with sub-1 V driving electronics

**Fig. 6:**
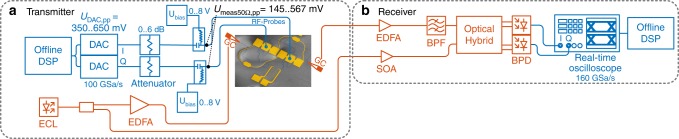
Experimental setup for low-drive voltage data generation experiment without driving amplifier

The experimental setup used during low drive voltage experiments is depicted in Fig. 6. It is similar to setup Fig. 5, however, the electrical driving amplifiers have been removed. Electrical 6 dB attenuators have been inserted to achieve sub-300 mV electrical drive voltages. The thermo-optic phase shifter has not been used during experiments without electrical driver amplifiers. The IQ operating point was adjusted by using the operating wavelength.

In the transmitter, electrical output swing voltages between 350 and 650 mV have been set. To measure the electrical drive voltage applied to the device, the bias tees have been removed from the RF-probes and connected to a real-time oscilloscope. Peak-to-peak voltages between 145 and 570 mV have been measured. Note, that the RF probes introduce additional losses of ~0.25 dB at 7 GHz and ~1.1 dB at 67 GHz. These additional RF losses are not taken into account.

### Offline digital signal processing (DSP)

Offline DSP for waveform generation and synchronization was applied. Before the actual measurements, the setup was calibrated in an electrical back-to-back configuration.

The electrical transmitter consisted of a 100 GSa s^−1^ DAC (Micram DAC4). The realization of different symbol rates was done by tuning the oversampling ratio in the transmitter DSP. To maximize the statistically relevance of our measurements, we always generated waveforms derived from randomly distributed bits with a length as close to the memory size of the DAC of 2^19^  samples as possible.

The optical receiver detected the in-phase and quadrature part of the waveform and sampled the signal with 160 GSa s^−1^. For the 100 GBd measurements, we applied a static digital post-distortion filter at the beginning of the DSP^73^. The following steps were timing synchronization, carrier recovery and linear and non-linear equalization. Finally, we measured the bit to error ratio (BER). The noise distribution of the synchronized symbols was used to estimate EVM and SNR^74^. In case of very few or no counted bit errors, the BER was estimated using the SNR.

### Temperature stability measurement

The chip was mounted on top of a thermo-electric heater with thermal paste. The surface temperature of the heater was measured using a PT1000 temperature sensor and set to the desired temperature by feeding an electrical current through the thermo-electric heater. For each measurement the DC bias of the individual MZMs was adjusted in a range between ±8 V, the IQ bias was adjusted using the on-chip thermo-optic phase shifter.

### Device fabrication

The plasmonic IQ modulators were fabricated in-house on a silicon-on-insulator (SOI) wafer consisting of a 220-nm-thick silicon device layer and a 3-μm-thick buried oxide. Electron-beam lithography using a Vistec EBPG5200 system was applied to pattern the structures in all process steps. A negative tone electron-beam resist was used to pattern photonic components comprising waveguides, MMIs and fiber-to-chip grating couplers (GCs)^69^. The patterns were transferred into the silicon device layer (fully etched structures) by applying an inductively coupled plasma—reactive ion etching (ICP-RIE). Partial etching (70 nm deep) of blazed GCs was realized with the use of a positive tone electron-beam resist and the ICP-RIE process as in the case of the fully etched silicon structures. A 700-nm-thick SiO_2_ cladding layer was deposited by plasma-enhanced chemical vapor deposition (PECVD) and locally opened in the active plasmonic sections using reactive ion etching (RIE). The SiO_2_ cladding reduces the influence of the OEO cladding on the GC performance. Plasmonic MIM waveguides and electrodes as well as the heater structure were produced with a lift-off process applied to a 150-nm-thick e-beam evaporated layer of gold. The organic electro-optic (OEO) material composite 75% wt HD-BB-OH/25% wt YLD124^59^ was deposited in a post-processing step by spin coating. Subsequently, a poling process induced the nonlinearity^38^. Envisioning co-integration with other devices, the OEO cladding material may be locally removed by an additional lithography step followed by structuring, e.g., by an O_2_ plasma-etching process.

## Supplementary information

Suplementary Information

## Data Availability

A comparison of state-of-the-art IQ modulators is provided in Supplementary Table 1. Information on the IQ modulators used in this work is provided in Supplementary Table 2. Detailed information on the measurements displayed in Fig. 2 are provided in Supplementary Table 3. Detailed information on the measurements performed with sub-1V driving electronics, are provided in Supplementary Table 4. Data of measurements displayed in Fig. 3 is provided in Supplementary Table 5. The datasets generated during and/or analysed during the current study are available from the corresponding author on reasonable request.
