# Supplementary material for: Plasmonic IQ modulators with attojoule per bit electrical energy consumption
Source: Nat Commun. 2019 Apr 12;10:1694. doi: 10.1038/s41467-019-09724-7 (PMC6461703; doi:10.1038/s41467-019-09724-7)
Supplement: Supplementary file 1 — Suplementary Information [file 41467_2019_9724_MOESM1_ESM.pdf]

Supplementary Information for:

Heni, Wolfgang et al.

Plasmonic IQ Modulators with Attojoule per Bit Electrical Energy Consumption

Supplementary Table 1: State-of-the-art IQ modulators

Technology	Fiber-to-fiber loss [dB]	Device loss [dB]	$U_{\pi}L$ [Vmm]	Active Length [mm]	Line rate [Gbit/s]	EO Bandwidth	Reference
**$LiNbO_3$**	~10	-	10-100	10-100	600	~40 GHz	[1,2]
**GaAs**	18	< 8 dB	90	30	150	~30 GHz	[3]
**Si**	~15	6.8 dB	30	4.5	350	~30 GHz	[4]
**InP**	9	~2 dB	~5	~3.6	448	~70 GHz	[5,6]
**SOH**	17.5*	8.5 dB	1	0.6	400	~40 GHz	[7]
**Plasmonic-Organic Hybrid**	19.2*	11.2	0.13	0.015	400	>500 GHz [8]	This work

* fiber-to-fiber losses can be reduced to <10 dB when relying on a dedicated fabrication process

Supplementary Table 2 Summary of investigated devices including fiber-to-fiber and on-chip losses.

Device	L_{active} [µm]	w_{slot} [nm]	On-chip loss [dB]	Photonic-plasmonic converter loss [dB]	Loss plasmonic section [dB]	Fiber-to-chip coupling loss [dB per coupler]	Fiber-to-fiber loss [dB]
**IQ1 621**	15	130	~11.2	~1.7	~7.8	~4	19.2
**IQ2 622**	20	130	~14.5	~1.7	~11.1	~4	22.5
**IQ3 626**	20	130	~13.7	~1.7	~10.3	~4	21.7

Supplementary Table 3 Measurements of Figure 2

Modulation	Device	$U_{meas50\Omega,pp}$ [V]	E_{bit} † [fJ bit^{-1}]	BER	BER without el. equalizer	P_{opt} [dBm]	$U_{IQ,pk}$ [V]
**50 GBd QPSK (100 Gbit s^{-1})**	IQ1	~2.0	10.2	6.86×10^{-8}*	3.3×10^{-4}	20	~2.0
	IQ2	1.92	12.4	4.96×10^{-6}ⴕ	5.9×10^{-4}ⴕ	15	1.92
	IQ3	1.92	12.4	8.25×10^{-23}*	1.9×10^{-9}*	18	1.92
**50 GBd 16QAM (200 Gbit s^{-1})**	IQ1	2.95	6.2	1.9×10^{-4}ⴕ	6.6×10^{-3}ⴕ	20	2.95
	IQ3	1.99	3.7	3.1×10^{-4}	6.8×10^{-3}	18	1.99
**100 GBd QPSK (200 Gbit s^{-1})**	IQ1	2.23	12.7	6.63×10^{-9}*	1.6×10^{-6}	18	2.23
	IQ2	3.45	40.0	1.26×10^{-6}*	5.2×10^{-5}	19	3.45
	IQ3	1.77	10.5	2.97×10^{-7}*	6.5×10^{-5}	18	1.77
**100 GBd 16QAM (400 Gbit s^{-1})**	IQ1	2.75	5.36	1.37×10^{-2}	6.3×10^{-2}	20	2.75
	IQ2	2.67	6.64	1.48×10^{-2}	6.4×10^{-2}	15	2.67
	IQ3	1.48	2.04	2.36×10^{-2}	4.5×10^{-2}	20	1.48

†Device capacitance of IQ1: 2.55 fF, for IQ2 and IQ3: 3.36 fF *BER estimated from SNR, no/not enough errors counted for statistical relevant measured BER.[9] ⴕMeasurement performed with uncalibrated transmitter, BER estimated from SNR.

Supplementary Table 4 Electrical energy consumption per bit at different symbol rates / modulation formats. All measurements performed with IQ3.

Modulation	$U_{meas50\Omega,pp}$	E_{bit}	BER	$U_{DAC,pp}$	$U_{IQ,pk}$	Attenuator
**25 GBd QPSK (50 Gbit s^{-1})**	145 mV	0.07 fJ bit^{-1}	2.0×10^{-3}	350 mV	145 mV	6 dB
	187 mV	0.12 fJ bit^{-1}	1.66×10^{-5}*	450 mV	187 mV	6 dB
**50 GBd QPSK (100 Gbit s^{-1})**	326 mV	0.36 fJ bit^{-1}	2.0×10^{-4}	350 mV	326 mV	0 dB
**50 GBd 16 QAM (200 Gbit s^{-1})**	567 mV	0.30 fJ bit^{-1}	2.0×10^{-2}	650 mV	567 mV	0 dB
**100 GBd QPSK (200 Gbit s^{-1})**	426 mV	0.61 fJ bit^{-1}	1.4×10^{-3}	650 mV	426 mV	0 dB
**100 GBd 16QAM (400 Gbit s^{-1})**	1.48 V	2.04 fJ bit^{-1}	2.36×10^{-2}	650 mV	1.48 V	RF Amplifier

* BER estimated from SNR, no/not enough errors counted for statistical relevant measured BER.[9]

Supplementary Table 5: Operation at elevated temperatures. Measurement parameters of measurements M1-M5 shown in Figure 3. All measurements at 100 Gbit s^{-1} (50GBd QPSK).

	Temp	Time at temp	Operating time	BER	EVM
	~22.5	>8h	7h	8.4×10^{-06}*┼	22.5 %┼
**M1**	42 °C	20 min	7.8 h	3.9×10^{-8}*	18.4 %
**M2**	65 °C	25 min	8.7 h	6.38×10^{-7}*	20.1 %
**M3**	65 °C	70 min	9.4 h	9.24×10^{-6}*	22.6 %
**M4**	75 °C	20 min	10 h	7.08×10^{-7}*	20.4 %
**M5**	75 °C	80 min	11 h	2.31×10^{-6}*	21.2 %

* BER estimated from SNR, no/not enough errors counted for statistical relevant measured BER.[9] ┼Measurement performed with uncalibrated transmitter.

Supplementary References

1 Schuh, K. *et al.* Single carrier 1.2 Tbit/s transmission over 300 km with PM-64 QAM at 100 GBaud. in *Optical Fiber Communication Conference.* Th5B. 5 (Optical Society of America).

2 Raybon, G. *et al.* Single-carrier all-ETDM 1.08-Terabit/s line rate PDM-64-QAM transmitter using a high-speed 3-bit multiplexing DAC. in *2015 IEEE Photonics Conference (IPC).* 1-2.

3 Schindler, P. C. *et al.* Monolithic GaAs electro-optic IQ modulator demonstrated at 150 Gbit/s with 64QAM. *Journal of Lightwave Technology* **32**, 760-765 (2014).

4 Lin, J., Sepehrian, H., Rusch, L. A. & Shi, W. CMOS-Compatible Silicon Photonic IQ Modulator for 84 Gbaud 16QAM and 70 Gbaud 32QAM. in *2018 Optical Fiber Communications Conference and Exposition (OFC).* 1-3.

5 Ogiso, Y. *et al.* Over 67 GHz bandwidth and 1.5 V Vπ InP-based optical IQ modulator with nipn heterostructure. *Journal of Lightwave Technology* **35**, 1450-1455 (2017).

6 Ogiso, Y. *et al.* Ultra-High Bandwidth InP IQ Modulator co-assembled with Driver IC for Beyond 100-GBd CDM. in *Optical Fiber Communication Conference.* Th4A. 2 (Optical Society of America).

7 Wolf, S. *et al.* Coherent modulation up to 100 GBd 16QAM using silicon-organic hybrid (SOH) devices. *Opt. Express* **26**, 220-232 (2018).

8 Burla, M. *et al.* 500 GHz plasmonic Mach-Zehnder modulator enabling sub-THz microwave photonics. *arXiv preprint arXiv:1901.00477* (2018).

9 Schmogrow, R. *et al.* Error vector magnitude as a performance measure for advanced modulation formats. *Ieee Photonic Tech L* **24**, 61-63 (2012).
